# Sedentary behaviour may cause differences in physical outcomes and activities of daily living in older cardiovascular disease patients participating in phase I cardiac rehabilitation

**DOI:** 10.1038/s41598-024-65001-8

**Published:** 2024-06-18

**Authors:** Kazuhiro P. Izawa, Kodai Ishihara, Yuji Kanejima, Masahiro Kitamura, Asami Ogura, Ikko Kubo, Koichiro Oka, Peter H. Brubaker, Hitomi Nagashima, Hideto Tawa, Daisuke Matsumoto, Ikki Shimizu

**Affiliations:** 1 Department of Public Health, Graduate School of Health Sciences, Kobe University, 10-2 Tomogaoka 7-Chome, Suma-ku, Kobe, 654-0142 Japan; 2 Cardiovascular Stroke Renal Project (CRP), Kobe, Japan; 3 Department of Physical Therapy, Faculty of Nursing and Rehabilitation, Konan Women’s University, Kobe, Japan; 4 Department of Rehabilitation, Kobe City Medical Center General Hospital, Kobe, Japan; 5 School of Physical Therapy, Faculty of Rehabilitation, Reiwa Health Sciences University, Fukuoka, Japan; 6 Department of Rehabilitation, Sanda City Hospital, Sanda, Japan; 7 Department of Rehabilitation, Yodogawa Christian Hospital, Osaka, Japan; 8 Faculty of Sport Sciences, Waseda University, Saitama, Japan; 9 Departments of Health and Exercise Science, Wake Forest University, Winston-Salem, NC USA; 10 Department of Rehabilitation, Shinyukuhashi Hospital, Yukuhashi, Japan; 11 Department of Cardiology, Sanda City Hospital, Sanda, Japan; 12 Department of Cardiovascular Medicine, Yodogawa Christian Hospital, Osaka, Japan; 13 Department of Diabetes, Sakakibara Heart Institute of Okayama, Okayama, Japan

**Keywords:** Activities of daily living, Daily life, Older cardiovascular disease patients, Phase I cardiac rehabilitation, Physical outcomes, Sedentary behaviour, Physiology, Environmental sciences

## Abstract

This study aimed to investigate the rate of sedentary behaviour and differences in physical outcomes and activities of daily living (ADL) based on sedentary behaviour time of hospitalized older cardiovascular disease patients undergoing phase I cardiac rehabilitation. Older cardiovascular disease patients were enrolled from October 2020 to September 2023 and were divided into the high sedentary behaviour group (≥ 480 min/day) and low sedentary behaviour group (< 480 min/day). Patients’ clinical characteristics, usual gait speed, and Five Times Sit to Stand Test time were compared as indices of physical outcomes. Motor, cognitive, and total Functional Independence Measure (FIM) scores were used as indices of ADL and compared between groups using analysis of covariance. Final analysis included 402 patients (mean age: 76.7 years, female: 35.3%). The high sedentary behaviour group included 48.5% of the study patients. After adjustment for baseline characteristics, gait speed (0.80 ± 0.27 vs. 0.96 ± 0.23 m/s, p < 0.001) was lower and FTSST time (11.31 ± 4.19 vs. 9.39 ± 3.11 s, p < 0.001) was higher in the high sedentary behaviour group versus low sedentary behaviour group. Motor (85.82 ± 8.82 vs. 88.09 ± 5.04 points, p < 0.001), cognitive (33.32 ± 2.93 vs. 34.04 ± 2.24 points, p < 0.001), and total FIM scores (119.13 ± 10.66 vs. 122.02 ± 6.30 points, p < 0.001) were significantly lower in the high sedentary behaviour group versus low sedentary behaviour group after adjustment. In older cardiovascular disease patients in phase I cardiac rehabilitation, sedentary behaviour time might influence physical outcomes and ADL at discharge. It is thus important to consider the amount of sedentary behaviour time spent by these patients during daily life while hospitalized.

## Introduction

Cardiovascular disease (CVD) affects a growing number of older patients worldwide, including those with heart failure (HF), ischaemic heart disease, and valvular disease^1–4^. Unfortunately, patients with CVD face high mortality rates and frequent readmissions that can lead to significant medical costs^1–8^. These patients often report low levels of physical activity that affect their health-related quality of life (HRQOL) and increase the risk of disease progression and mortality^5–8^.

To promote physical activity, recent research has highlighted the health risks associated with sedentary behaviour (SB), defined as any behaviour while awake that results in an energy expenditure of 1.5 metabolic equivalents or less when an individual is sitting, reclining, or lying down^9,10^. The risks of death, obesity, diabetes, and CVD are increased in those with SB^9,10^.

Hospitalized patients often spend most of their time in bed or sitting, which reduces physical activity. Various comorbidities are associated with lower daily step counts and less time spent upright^11–16^. Additionally, non-lying time is a factor contributing to hospital-associated functional decline (HAFD) in older patients undergoing transcatheter aortic valve implantation (TAVI)^17^. Nationwide registry surveys in Japan indicate that 7.4% to 37.1% of older HF patients experience HAFD, underscoring the need for convalescent rehabilitation^8,18^.

To improve health outcomes in older CVD patients, promotion of physical activity and reduction of SB time are crucial. By focussing on SB, healthcare workers can enhance the cardiovascular health and overall well-being of this vulnerable population^19,20^.

The Asian Working Group for Sarcopenia (AWGS) 2019 has recommended measurements of gait speed (GS) and Five Time Sit-to-Stand Test (FTSST) times to screen older populations and older CVD patients^21–23^. These assessments are suitable for evaluating physical performance and sarcopenia as neither test requires specialized procedures or skills^21–23^.

The Functional Independence Measure (FIM) is a widely recognized indicator worldwide for assessing activities of daily living (ADL) in older CVD patients^24,25^. Notably, the motor FIM score has been identified as an independent predictor of re-hospitalization within 90 days^25^. We used the GS and FTSST in the present study to assess physical outcomes and the FIM to evaluate ADL performance when screening SB time.

Because SB is a crucial target that may predict improvements in cardiac knowledge gained from patient education programs administered in phase I and II CR^20,26–28^, assessment of SB, especially in patients with high SB, is essential in both CR phases. This approach ensures that patients better comprehend and consider the educational content they receive during CR.

Currently, few reports have addressed high SB time and differences in physical outcomes and ADL based on SB in older CVD patients undergoing phase I CR. We hypothesized that there would be differences in physical outcomes and ADL between patients with high and low SB and that older CVD patients with high SB would have poorer physical outcomes and ADL than those with low SB in phase I CR. Thus, the purpose of this study was to investigate the rate of SB and differences in physical outcomes and ADL based on SB time in older CVD patients in phase I CR.

## Methods

### Study design

This prospective multicentre cohort study (The Kobe-Cardiac Rehabilitation project for people around the World: K-CREW) included patients from four affiliated hospitals with 200 to 580 beds that conduct phase I CR^28^. Included patients were those admitted to the affiliated hospitals from October 2020 to September 2023, hospitalized for a period of more than 5 days, and participated in phase I CR. Patients with CVD admitted only for therapeutic interventions such as coronary angiography, percutaneous coronary intervention without CR, cardiac ablation, and pacemaker battery replacement were excluded. Patients with probable dementia (based on diagnosis or Mini-Mental State Examination score < 24), and those with difficulty in walking alone, who refused to provide informed consent, had missing data, or who died in hospital were also excluded.

### Comprehensive phase I CR

Comprehensive phase I CR was conducted following the guidelines of the Japanese Circulation Society for rehabilitation of patients with CVD^1,2^. Phase I CR started no later than three days after admission and cardiac surgery^1,2,28^. Exercise was performed 5 to 7 days per each week of CR at an intensity ranging from 11 to 13 on the Borg scale or just below the patient’s anaerobic metabolism threshold. Exercise times included 10 to 20 min for upper and lower body stretching, < 25 min for aerobic exercise including warm-up, aerobics, and cool-down exercises, and 10 to 20 min for resistance training according to previous reports^1,2,6,16,28^. Exercise training was adjusted according to each patient’s condition.

The older CVD patients were instructed about their disease, lifestyle choices, nutrition improvement, medications, and exercise by a medical doctor, nurse, registered dietitian, pharmacist, and physical or occupational therapists, respectively^1,2,6,16,28^.

### Patient clinical characteristics

Clinical characteristics of age, sex, body mass index (BMI), employment, living together, smoking, marriage, main diagnosis, left ventricular ejection fraction, Charlson Comorbidity Index^29^, levels of serum haemoglobin and creatinine, and medications at the time of admission were collected from the medical records by one researcher. The patients were also asked to answer a cognitive function questionnaire (Mini Mental State Examination) at discharge.

### Sedentary behaviour time

We used the Workforce Sitting Questionnaire^30^ to evaluate SB time. This questionnaire, whose reliability and validity have already been confirmed in Japan^31^, includes six items related to SB time over 1 week that assess driving, transportation, work, television viewing, personal computer/smartphone use, and other leisure time activity^28,31^. As the older CVD patients were hospitalized, 0 min were assigned to the items of driving and transportation times^28,31^. Each item was answered for workdays and non-workdays over a 7-day period. After the questionnaires were collected by a researcher, total SB time in min/day as it related to the six items over the 7-day period was calculated as total SB time in minutes ÷ 7 days^28,31^. Therefore, SB time measured for the entire day was considered to indicate SB. We then divided the patients into two groups according to a cutoff value for SB time of 480 min/day (low SB group: < 480 min/day and high SB group: ≥ 480 min/day) as described in a previous study^28,32^.

### Gait speed and Five Times Sit to Stand Test to assess physical outcome

GS and the FTSST were used as indices of physical outcome in the present study. Usual GS was measured over a 4-m distance on a flat floor with a total pathway length of 6 m^28^. We instructed each participant to walk the 6-m distance as fast as possible without falling. The time taken to walk the 4-m distance in the middle of the 6-m-long walking path was measured using a stopwatch. The measurement was conducted twice and the shorter time was used^21–23,28^. The FTSST requires the patients to cross their arms across their chest, stand up from a chair, and return to a seated position five times as quickly as possible. AWGS 2019 considers GS < 1.0 m/s and FTSST ≥ 12 s as criteria for low physical performance indicative of sarcopenia^23^.

### Motor, cognitive, and total FIM for ADL

The primary outcome measure for ADL was the FIM score^33,34^. The FIM comprises 18 items, each scored on a scale of one to seven points. The total FIM score ranges from 18 to 126 points and reflects the level of dependence in actual ADL. The FIM is divided into the motor domain, which includes 13 items related to physical movements, such as self-care, sphincter control, transfers, and locomotion, and the cognitive domain, consisting of five items for assessment of communication and social cognition. A higher FIM score indicates greater independence in ADL. At discharge, one or three CR staff members assessed each patient’s GS, FTSST, and FIM results.

### Statistical analysis

Results are expressed as the mean ± standard deviation (SD). We used parametric and chi-square tests to analyse differences between the high SB and low SB groups. Additionally, we used an unpaired *t*-test to assess differences in clinical characteristics between the two patient groups. To compare GS, FTSST, and motor, cognitive, and total FIM values between the groups, we performed a one-way analysis of covariance (ANCOVA) with relevant variables as covariates. These covariates were selected based on significant differences observed in clinical characteristics between the two groups using chi-square analyses and *t*-tests. A p-value < 0.05 was considered statistically significant. For our analyses, we used IBM SPSS 29.0 statistical software^35^, which was developed by IBM SPSS Japan, Inc.

### Ethical approval

This study was approved by the Institutional Review Board for Ethics at the Graduate School of Health Sciences, Kobe University (Approval No. 951-2), and each affiliated hospital received approval from its local ethics committee.

### Informed consent

Informed consent was obtained from each patient.

## Results

### Flow chart of study participants

**Figure 1:**
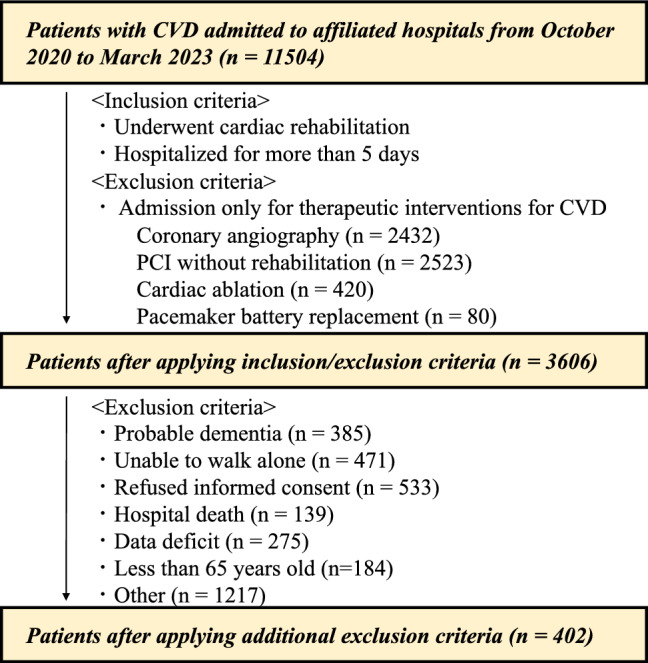
Flow chart of the study participants. *CVD* cardiovascular disease, *PCI* percutaneous coronary intervention.

Among 11,504 CVD patients admitted to the affiliated hospitals during the study period, 3606 patients met the inclusion criteria, including being hospitalized for more than 5 days and undergoing phase I CR. After excluding 3204 patients who met the exclusion criteria, 402 patients (mean age: 76.7 years, female: 35.3%) were finally included. A flow chart of the study participants is shown in Fig. 1.

### Patient characteristics between the high SB and low SB groups

Clinical characteristics of the patients between the high SB group and low SB group can be compared in Table 1, which shows that the high SB group comprised 48.5% (195 of 402) of the older CVD patients. There were significant differences in age, employment, main diagnosis, and SB time between the two groups.

**Table 1:** Clinical characteristics of the patients in the high SB and low SB groups.

Characteristic	High SB (n = 195)	Low SB (n = 207)	t** or χ^2^ value	p-Value
Age (years)*	78.3 ± 7.6	75.2 ± 6.7	4.36**	< 0.001
Sex, female (%)	75 (38.5)	67 (32.4)	1.63	0.201
Body mass index (kg/m^2^)*	21.9 ± 3.7	22.4 ± 3.5	− 1.29**	0.195
Employment (%)	45 (23.1)	105 (50.7)	32.81	< 0.001
Living together (%)	163 (83.6)	160 (77.3)	2.52	0.112
Smoking (%)	104 (53.3)	91 (44.0)	3.53	0.060
Marriage (%)	131 (67.2)	144 (69.6)	0.26	0.607
Main diagnosis (%)			46.87	< 0.001
Heart failure	107 (54.8)	47 (22.7)		
Ischaemic heart disease	75 (38.5)	121 (58.4)		
Valvular disease	5 (2.6)	14 (6.8)		
Others	8 (4.1)	25 (12.1)		
Left ventricular ejection fraction (%)*	51.9 ± 15.2	51.7 ± 12.7	0.14**	0.88
Charlson Comorbidity Index*	2.4 ± 2.8	2.3 ± 2.2	0.27**	0.785
Haemoglobin (g/dL)*	12.4 ± 2.3	12.4 ± 2.2	− 0.19**	0.85
Creatinine (mg/dL)*	1.4 ± 1.7	1.3 ± 1.1	1.42**	0.16
Blood urea nitrogen (mg/dL)*	25.1 ± 13.6	23.3 ± 14.3	1.33**	0.18
Medications (%)				
Beta blocker (%)	130 (66.7)	143 (69.1)	0.27	0.604
ACE-I and ARB (%)	99 (50.8)	111 (53.6)	0.32	0.567
Diuretic (%)	124 (63.6)	116 (56.0)	2.38	0.123
Statin (%)	122 (62.6)	121 (58.5)	0.71	0.400
SB time (min/day)*	718.42 ± 152.3	240.1 ± 118.7	35.22**	< 0.001

*SB* sedentary behaviour, *ACE-I* angiotensin-converting enzyme inhibitor, *ARB* angiotensin II receptor blocker.

*Mean ± standard deviation. **t value.

### Assessments of physical outcome between the high SB and low SB groups

**Figure 2:**
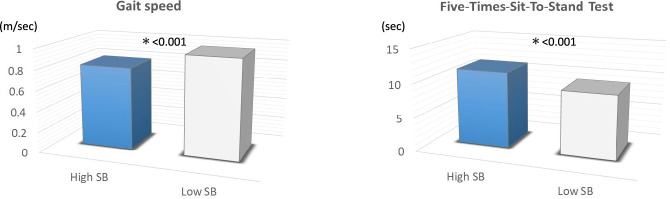
Comparison of the physical outcomes of GS and FITTS between the high SB group and low SB group. *FITTS* Five Times Sit to Stand Test, *GS* gait speed, *SB* sedentary behaviour.

The results showed that GS (0.80 ± 0.27 vs. 0.96 ± 0.23 m/s, p < 0.001) was significantly lower and FTSST time (11.31 ± 4.19 vs. 9.39 ± 3.11 s, p < 0.001) was significantly higher in the high SB group versus low SB group after adjusting for clinical characteristics (Table 2, Fig. 2).

**Table 2:** Physiological outcomes and ADL in the high SB and low SB groups.

Values	High SB	Low SB	F value	p-value
Physiological outcomes				
GS (m/s)*	0.80 ± 0.27	0.96 ± 0.23	111.60	< 0.001
FTSST (s)*	11.31 ± 4.19	9.39 ± 3.11	58.22	< 0.001
ADL				
Motor FIM (points)*	85.82 ± 8.82	88.09 ± 5.04	49.99	< 0.001
Cognitive FIM (points)*	33.32 ± 2.93	34.04 ± 2.24	19.44	< 0.001
Total FIM (points)*	119.13 ± 10.66	122.02 ± 6.30	52.20	< 0.001

*SB* sedentary behaviour time, *GS* gait speed, *FTSST* Five-Times-Sit-To-Stand Test, *ADL* activities of daily living, *FIM* Functional Independence Measure.

*Mean ± standard deviation. Adjusted for baseline characteristics.

### Motor, cognitive, and total FIM scores for ADL between the high SB and low SB groups

**Figure 3:**
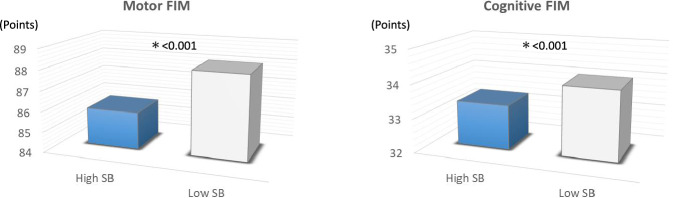
Comparison of motor and cognitive FIM scores for activities of daily living between the high SB group and low SB group. *FIM* Functional Independence Measure, *SB* sedentary behaviour.

**Figure 4:**
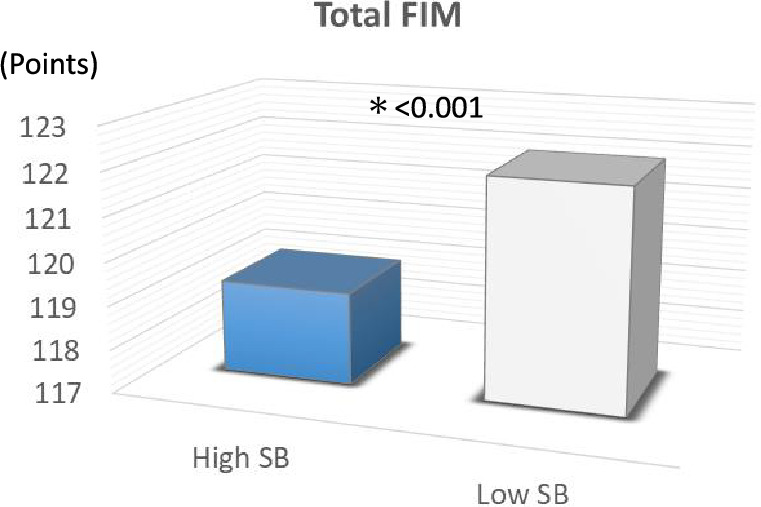
Comparison of total FIM scores for activities of daily living between the high SB group and low SB group. *FIM* Functional Independence Measure, *SB* sedentary behaviour.

Motor (85.82 ± 8.82 vs. 88.09 ± 5.04 points, p < 0.001), cognitive (33.32 ± 2.93 vs. 34.04 ± 2.24 points, p < 0.001), and total FIM scores (119.13 ± 10.66 vs. 122.02 ± 6.30 points, p < 0.001) were significantly lower in the high SB group versus low SB group after adjusting for clinical characteristics (Table 2, Figs. 3 and 4).

## Discussion

This study examined the differences in physical outcomes and ADL among older CVD patients based on their SB time. Although several previous studies have investigated the impact of SB time and/or physical activity in hospitalized patients^9,15,16^, the present study specifically focused on older CVD patients. We investigated the cutoff values of SB time based on methods used in previous studies^28,32^. In a recent study in Japan, Kono et al. reported that non-lying time could be one of the associated factors of HAFD in older patients undergoing TAVI^17^. They suggested that non-lying time of about 480 min (8 h) per day during hospitalization may be an initial target for preventing HAFD. Therefore, on the basis of this previous study, we considered this cutoff value used for SB time to be appropriate.

Cattanach et al. previously reported that patients were observed to be in bed 51% of the time and sitting out of bed 43%, standing 1%, and walking 5% of the time. The response to a questionnaire they used indicated that one third of the participants were not observed to walk during the observation period, and, moreover, 38% of the participants had expected to remain in bed while in hospital^15^. Even though the assessment methods between their study and ours were different, the present study showed a high rate of SB of 48.5%. Our previous study^28^, which included both middle-aged and older patients, showed a high rate of SB of 47.6%. However, the rate of SB was higher in the present study as it was limited only to older patients. In other previous studies, although diseases and ages of the study patients were different, the older patients tended to engage in more sitting SB time and spend more time in bed^13,14^. In their systematic review, Harvey et al.^36^ suggested that older adults (age ≥ 60 years) are one of the most sedentary groups in society, spending on average 80% of their time in a seated posture with 67% being sedentary for more than 8.5 h per day. In the present study, the total sitting SB time in the high SB group was 718.42 min/day, i.e., approximately 11.97 h/day. Because SB occurred primarily during leisure time, outside of phase I CR, the greatest reduction in total SB time of older CVD patients with high SB can likely be achieved by targeting their leisure time sedentary activities during hospitalization. We consider it important to address this problem beginning in phase I CR because as Chastain et al.^37^ reported, the effectiveness of interventions to reduce SB in community-dwelling older adults remains unclear.

There were significant differences in the characteristics of age, employment, and main diagnosis between the patients in the two study groups. Notably, these factors largely align with predictors of high SB levels in the general population and of several diseases^9,20,26,27,38,39^. Our findings suggest that these factors may also impact high SB levels in older CVD patients.

With regard to physical outcomes, GS was lower and FTSST time was higher (Table 2, Fig. 2) in the high SB versus low SB group after adjusting for baseline characteristics. Individuals with a GS below 1.0 m/s are at high risk for leg injury, hospitalization, and death^22,40^. In the programming of pedestrian traffic signals in Japan, a GS of 1.0 m/s or faster is required to cross the street within the programmed period^22^. Thus, having a GS of 1.0 m/s or faster is an important ability needed for various public activities such as shopping, hobbies, and work after discharge from hospital. The value was close to 1.0 m/s for the low SB group but was much lower than 1.0 m/s for the high SB group.

The FTSST time in the high SB group was 11.31 s, similar to the low physical performance value indicated in AWGS 2019 (≥ 12 s)^23^. In addition, Camarzana et al. recently suggested that the FTSST was an independent predictor of 1-year mortality in patients with severe aortic stenosis who did not undergo valve replacement (59 patients; age, 86.1 years)^41^. Therefore, the reserve capacity for GS in the high SB group is low, and FTSST time is high, indicating that special attention should be paid to older CVD patients.

Motor, cognitive, and total FIM scores were significantly lower in the high SB group compared to the low SB group (Table 2, Figs. 3 and 4). A previous study aimed to identify predictive factors for ADL as assessed by FIM at discharge in older heart failure patients with preserved ejection fraction. The receiver operating characteristic curves yielded cutoff values for predicting ADL at discharge of 34.5 points for the motor FIM score and 28.5 points for the cognitive FIM score. Notably, the motor and cognitive FIM scores in the high SB group of the present study included patients with scores above these reported thresholds, potentially minimizing the impact of SB on discharge results. However, other previous studies have suggested that FIM scores at discharge serve as independent predictors of re-admission and mortality at 90 and 180 days in CVD patients^25,42^. Kitamura et al. further classified older heart failure patients into four groups based on a previous study’s cutoff values for the Geriatric Nutritional Risk Index (GNRI), an index of nutrition, and motor FIM. They reported that the rate of readmission avoidance was significantly lower in the group with GNRI < 92 and motor FIM < 75 within 90 days of discharge^25^.

Iwata et al. also investigated the prognostic impact of the FIM score on clinical outcomes in hospitalized patients with acute decompensated heart failure. They retrospectively analysed 473 patients with available pre-discharge FIM scores admitted to their institution. The primary outcome measures, defined as a composite of 180-day all-cause deaths and readmissions, were compared among three tertiles^42^. The median total FIM score was 102 (interquartile range: 85–115). Tertile 1 corresponded to an FIM score > 111, Tertile 2 to that of 90–111, and Tertile 3 to that of < 90. Even after multivariable adjustment, the results remained significant [Tertile 1 vs. 3: adjusted hazard ratio, 3.28; Tertile 2 vs. 3: 2.32]. FIM scores were significantly associated with readmission or death within 180 days of discharge in these hospitalized heart failure patients^42^.

Evaluation of FIM scores at the time of discharge, especially in relation to high SB, is crucial. Additionally, prognosis after discharge must be considered and measures implemented with a view to continuing phase II CR. Although our study focused on phase I CR, it is worth noting that in a different context (long-term acute care hospitals for patients after stroke), changes in FIM scores associated with the minimal clinically important difference were 22 points for total FIM, 17 points for motor FIM, and 3 points for cognitive FIM scores^43^. Although we did not specifically examine improvement in pre- versus post-FIM scores during phase I CR, ADL of the high SB group were significantly lower at discharge. Thus, older CVD patients with high SB are likely to experience not only loss of motor function but also a decline in cognitive function related to ADL in the future. It may be necessary for staff in charge of phase I CR to intervene for patients with SB during hospitalization to improve their ADL.

Our study also showed that high age, heart failure, and unemployment tended to be associated with high SB. As mentioned earlier, even though hospitalized patients with acute heart failure infrequently experience hospital-associated disability, only 44% of these patients were accepted into phase I CR^8^. Therefore, greater attention should be paid to these factors during phase I CR. Early mobilization to reduce SB, early screening for sarcopenia, and implementing aerobic exercise and resistance training during hospitalization are essential components of phase I CR.

The recent systematic review by Chastin et al. suggested that it remains unclear whether interventions to reduce SB are effective in decreasing sedentary time in community-dwelling older adults^37^. Furthermore, the impact of these interventions on the physical and mental health of community-dwelling older adults remains uncertain. Thus, further research is necessary to explore interventions aimed at reducing high SB and improving both physical outcomes and ADL of older CVD patients during phase I CR.

### Limitations

The present study has several limitations. First, the study is constrained by its small sample size and the limited number of female patients, which hindered the assessment of sex-related differences. Second, many of the older CVD patients were hospitalized solely for therapeutic interventions, such as pacemaker battery replacement, and were therefore excluded from this study, which may have introduced selection bias. Third, the study included various CVDs as the primary diagnosis, along with comorbidities requiring different treatments. Fourth, a self-reported subjective questionnaire was used to assess SB time, and the study lacked objective measurements made by a versatile device with various features and tools. Fifth, we could not investigate the relationship between SB time and prognosis, including re-admission rates and mortality. Notably, SB has been linked to acute detrimental effects on vascular function, blood pressure, and lipid levels, which may contribute to the risk of cardiovascular events and mortality^26,27,44^. Finally, it might be more insightful to report a comparison between values at baseline and the end of phase I CR to investigate effects of the multidisciplinary intervention on SB and how SB could be affected in both groups (e.g., would most inactive patients benefit from the intervention, or would they be more reluctant to participate in such a program in the first place?). In future trials, we will need to investigate differences in disease severity as they relate to SB to determine whether specific forms of intervention should be applied based on this analysis.

## Conclusions

This study investigated the impact of SB on physical outcomes and ADL of hospitalized older CVD patients undergoing phase I CR. We found that the high SB group had lower GS and longer FTSST times compared to the low SB group. Additionally, motor, cognitive, and total FIM scores for ADL were significantly lower in the high SB group, even after adjusting for other associated factors. These findings suggest that SB time may influence physical outcomes and ADL at discharge of older CVD patients participating in phase I CR. Therefore, it is essential to consider levels of SB during CR and encourage older CVD patients to engage in more physical activity while in hospital (Supplementary Fig. 1).

### Supplementary Information

Supplementary Figure 1.

## Data Availability

The datasets used and/or analysed in the current study are available from the corresponding author on reasonable request.
