# Supplementary material for: Sedentary behaviour may cause differences in physical outcomes and activities of daily living in older cardiovascular disease patients participating in phase I cardiac rehabilitation
Source: Sci Rep. 2024 Jun 18;14:14037. doi: 10.1038/s41598-024-65001-8 (PMC11189532; doi:10.1038/s41598-024-65001-8)
Supplement: Supplementary file 1 — Supplementary Figure 1. [file 41598_2024_65001_MOESM1_ESM.pptx]

## Slide 1
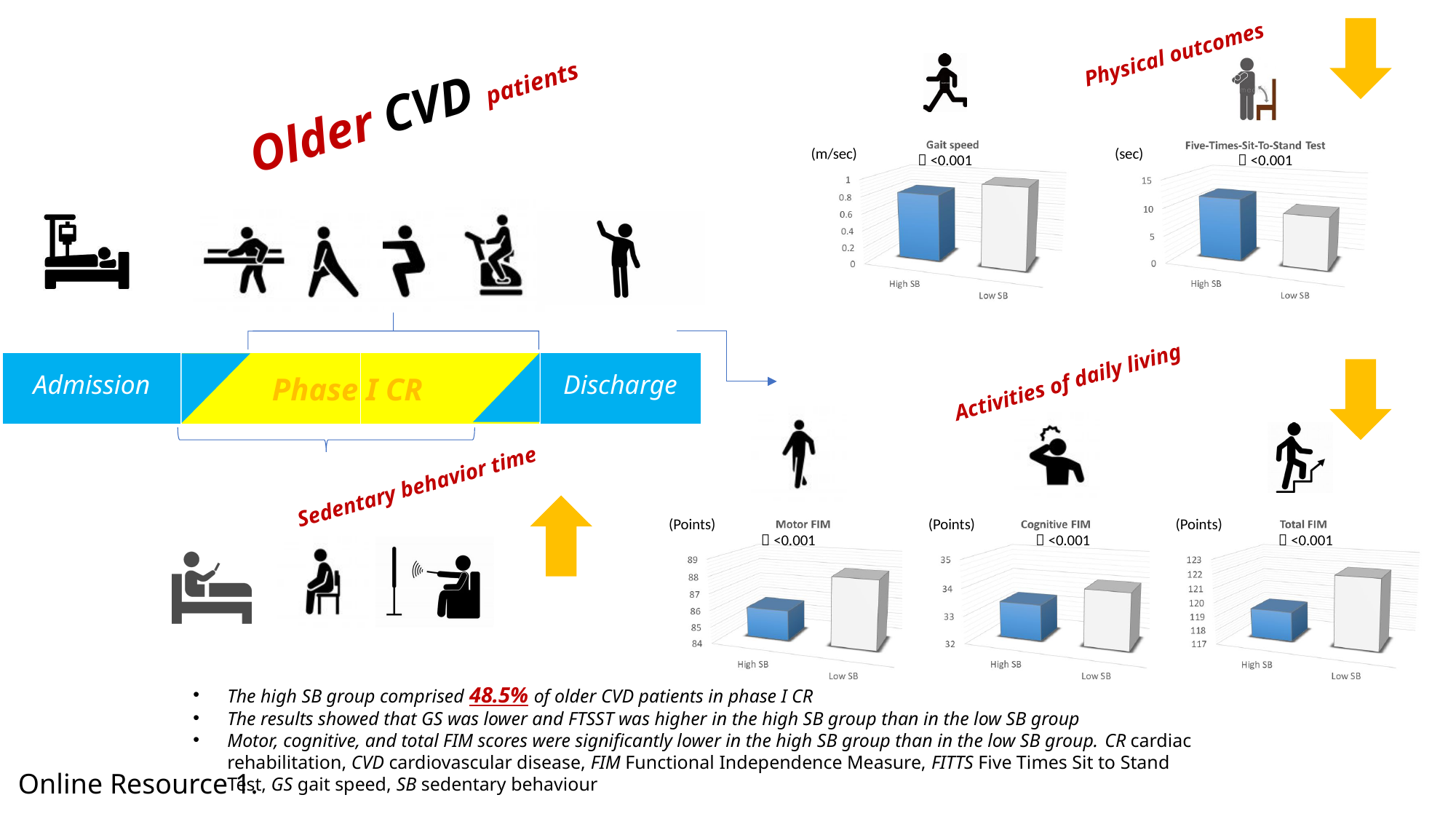

Physical outcomes
Older CVD patients
(m/sec)
(sec)
＊<0.001
＊<0.001
| Admission | | | Discharge |
| --- | --- | --- | --- |
Activities of daily living
Phase I CR
Sedentary behavior time
(Points)
(Points)
(Points)
＊<0.001
＊<0.001
＊<0.001
The high SB group comprised 48.5% of older CVD patients in phase I CR
The results showed that GS was lower and FTSST was higher in the high SB group than in the low SB group
Motor, cognitive, and total FIM scores were significantly lower in the high SB group than in the low SB group. CR cardiac rehabilitation, CVD cardiovascular disease, FIM Functional Independence Measure, FITTS Five Times Sit to Stand Test, GS gait speed, SB sedentary behaviour
Online Resource 1.
